# Venom Immunotherapy: From Proteins to Product to Patient Protection

**DOI:** 10.3390/toxins13090616

**Published:** 2021-09-01

**Authors:** Martin Feindor, Matthew D. Heath, Simon J. Hewings, Thalia L. Carreno Velazquez, Simon Blank, Johannes Grosch, Thilo Jakob, Peter Schmid-Grendelmeier, Ludger Klimek, David B. K. Golden, Murray A. Skinner, Matthias F. Kramer

**Affiliations:** 1 Allergy Therapeutics (UK) Ltd., Worthing BN14 8SA, UK; FeindorM@bencard.com (M.F.); matthew.heath@allergytherapeutics.com (M.D.H.); Simon.Hewings@AllergyTherapeutics.com (S.J.H.); Thalia.CarrenoVelazquez@AllergyTherapeutics.com (T.L.C.V.); Murray.Skinner@allergytherapeutics.com (M.A.S.); 2 Bencard Allergie GmBH, 80804 Munich, Germany; 3 Center of Allergy and Environment (ZAUM), School of Medicine and Helmholtz Center Munich, Technical University of Munich, 85764 Munich, Germany; simon.blank@helmholtz-muenchen.de (S.B.); johannes.grosch@helmholtz-muenchen.de (J.G.); 4 Experimental Dermatology and Allergy Research Group, Department of Dermatology and Allergology, University Medical Center Giessen and Marburg, Justus-Liebig-University Gießen, 35390 Giessen, Germany; Thilo.Jakob@derma.med.uni-giessen.de; 5 Allergy Unit, Department of Dermatology, University Hospital of Zürich, 8091 Zürich, Switzerland; peter.schmid@usz.ch; 6 Center for Rhinology and Allergology, 65183 Wiesbaden, Germany; Ludger.Klimek@Allergiezentrum.org; 7 Chesapeake Clinical Research, Baltimore, MA 21236-5992, USA; dbkgolden@gmail.com

**Keywords:** venom, VIT, wasp venom, honeybee venom, allergy, Hymenoptera, sensitization, adjuvant

## Abstract

In this review, we outline and reflect on the important differences between allergen-specific immunotherapy for inhalant allergies (i.e., aeroallergens) and venom-specific immunotherapy (VIT), with a special focus on Venomil^®^ Bee and Wasp. Venomil^®^ is provided as a freeze-dried extract and a diluent to prepare a solution for injection for the treatment of patients with IgE-mediated allergies to bee and/or wasp venom and for evaluating the degree of sensitivity in a skin test. While the materials that make up the product have not changed, the suppliers of raw materials have changed over the years. Here, we consolidate relevant historical safety and efficacy studies that used products from shared manufacture supply profiles, i.e., products from Bayer or Hollister–Stier. We also consider the characterization and standardization of venom marker allergens, providing insights into manufacturing controls that have produced stable and consistent quality profiles over many years. Quality differences between products and their impacts on treatment outcomes have been a current topic of discussion and further research. Finally, we review the considerations surrounding the choice of depot adjuvant most suitable to augmenting VIT.

## 1. Introduction

The immune responses to insect stings, such as those from wasps or bees in Hymenoptera venom allergies (HVA), can lead to severe and life-threatening reactions. These significantly impair the quality of life of patients with venom anaphylaxis [1,2,3]. HVA seems to be increasing in Europe [4,5]. Venom-specific immunotherapy (VIT) involves the repeated administration of allergens in order to modulate the immune response of the allergic individual. It is the only available treatment that results in complete prevention of severe allergic reactions—including disease modifying and/or long-term efficacy, or even curing of the allergy [6,7,8].

Common allergy-relevant species of Hymenoptera include those found within the superfamilies of Apoidea (honeybees; *Apis mellifera*) and of Vespoidea (yellow jackets; *Vespula* spp.), which are found all over the world, but particularly in the northern hemisphere [9,10,11,12]. Wasps of the cosmopolitan genus *Polistes* are also of great importance in the USA and Southern Europe [9].

Sensitization profiles against a complex mix of major and minor allergens from venoms can vary greatly between individuals. VIT is conducted with venom extracts derived from natural source materials (insect venom). Each manufacturer of VIT preparations is likely to have differences in down-stream processing to purify and formulate the venom extracts for VIT. Indeed, processing may result in differences in allergen composition, as shown in recent studies studying the component resolutions of therapeutic grade honeybee venom (HBV) extracts to examine cases of treatment failures [12,13,14,15,16,17,18]. In these instances, underrepresentation of relevant allergens has been highlighted, emphasizing the importance of a well-characterized allergen product [19].

Although VIT is often regarded as the role model of allergen-specific immunotherapy (AIT), there are important differences to consider. Insect venom allergy often involves life-threatening symptoms of anaphylaxis, whereas reactions against inhaled allergens only rarely cause fatality. Further, the natural means of allergen exposure is injection via the skin in the case of HVA and via mucosal surfaces in the case of allergic rhinitis and allergic asthma. Similarly, there are substantial differences between VIT and AIT against inhalant allergens:

- The route of administration of VIT is subcutaneous (s.c.) only, whereas in AIT aeroallergens can be administered s.c. but also sublingually.
- Standard VIT involves a 100 µg bee or wasp maintenance dosage, whereas uniform dosing recommendations do not exist for AIT (i.e., inhalant allergens) between comparable products.
- Aqueous and depot adjuvant formulations of VIT are marketed throughout Europe, whereas aqueous extracts for s.c. AIT with inhalant allergens are rarely used in Europe.
- Contraindications against VIT differ from those of AIT against inhalant allergens; consider the life-threatening nature of HVA [20].
- Success rates of VIT are substantially higher compared to AIT with inhalant allergens. However, this involves longer treatment courses of VIT (5 years standard VIT versus 3 years standard AIT with inhalant allergens) and high-dose VIT.
- Measuring success rates in VIT and AIT with inhalant allergens (e.g., primary outcome parameters in controlled clinical trials) differs substantially.
- About 30% of patients treated for HVA require life-long VIT. This group includes patients suffering from co-morbidities such as mastocytosis, patients with severe initial systemic sting reactions, patients who have had systemic adverse events during VIT, but also individuals with high risks of future bee and/or wasp stings, such as beekeepers or workers in confectioneries [21,22,23,24,25].

Thus, although VIT is a form of AIT, there are substantial differences between VIT and AIT for aeroallergens worth discussing/dissecting.

There are a number of companies throughout the world that manufacture and supply Hymenoptera venom extracts for clinical applications. The Venomil^®^ bee and/or wasp products (known hereon in as Venomil^®^) are provided as freeze-dried allergen extracts and a diluent to prepare a solution for injection.

While a number of venom immunotherapy products have existed for the last 50 years, the nature of allergen extraction and onward processing has a limited scope for change, as the products are typically purified extracts of allergens produced with a lyophilization stage. For products currently available, the allergen contents and excipient make-up remain broadly similar, though some have optimized processes to maintain the stability of their allergens, and others have developed different posology. The clinical evidence that supports many of these products dates back to the time when they were originally developed (i.e., Bayer or Hollister–Stier) and the evolution of product ownership under different organizations (Table 1). Venomil^®^ has been in active clinical use since the early 1980s, and the market authorizations are shown in Table 1.

VIT products provided by all companies are based on broadly similar, naturally-derived source materials. The processing steps associated with allergen-products are relatively straightforward. Source material is solubilized by extraction and filtration. The testing standards set by European and US health authorities are highly comparable. For example, the Guideline on Allergen Products: Production and Quality Issues provides guidance on the establishment and use of in-house reference preparations (IHRP) for quality control, including the analysis of batch-to-batch consistency. Additionally, criteria for the preparation of the serum pools used for potency measurements are defined. Furthermore, both European and US requirements include standardization of venoms for phospholipase and hyaluronidase enzymatic activity [26]. While different extraction and downstream processing conditions (such as temperature or buffer conditions) can produce a degree of allergen variability in the final product, such guideline requirements ensure a degree of consistency across batches. This review is the first to consolidate relevant historical safety and efficacy studies that included products with shared manufacturer supply profiles, i.e., venom products from Bayer or Hollister–Stier, and consider quality differences between products and the impacts on treatment outcomes.

## 2. Production of Venom Extracts

In the Venomil^®^ wasp (yellow jacket) venom product, *Vespula* spp. can consist of the following species: *V. germanica*, *V. maculifrons*, *V. pensylvanica*, *V. alascensis* and *V. squamosa.* A minimum of four different species of the genus *Vespula* are used in equal quantities. These are considered a suitable panel of species based on their level of characterization and homology [9]. Of note, *V squamosa* has a degree of antigenic activity considered unique, which formed the basis of justification by the FDA in 1980 to be specified by manufacturers [27,28,29].

The necessary quantities of frozen worker insects are thawed just prior to the dissection of venom sacs. Dissection is a critical manual operation requiring competency training and dexterity of the operators who remove the sting apparatus by grasping it with micro-forceps and pulling it along with the complete/intact venom sac away from the insect abdomen. Sac fragments are removed by filtration, once the venom sac is separated from associated structures. This process is repeated until the required number of aliquots for the specified batch size is completed. Meanwhile, aliquots containing venom sacs are stored frozen until the dissection process is finished. The frozen aliquots of venom sacs in extraction medium are thawed and transferred to an appropriate container immediately prior to further processing, and the extracted venom is kept cool in an appropriate buffer.

Honeybee venom is extracted by electro-stimulation of live bees (*Apis* spp.). The venom is air dried, collected and supplied (frozen) with a certificate of analysis.

During the extraction stage, *Vespula* venom sacs or freeze-dried bee venom is homogenized with an appropriate extraction medium and centrifuged, and then the extract (supernatant) is collected. The material is clarified through a 0.2 µm sterilizing filter to remove particulates (including wasp venom sac fragments) that may be present due to the venom extraction process and to control bioburden (microbial limit testing). The temperature is controlled during the clarification step. Bioburden is measured immediately prior to sterile filtration in accordance with GMP requirements for sterile products. The venom extract must be handled using aseptic techniques within a classified Grade A environment, as the drug substance is not terminally sterilized. The filtered venom extract is poured into sterile vials under Grade A conditions, and sterile lyophilization stoppers are partially seated on the vials prior to beginning the freeze-drying process, also in a Grade A environment.

**Figure 1:**
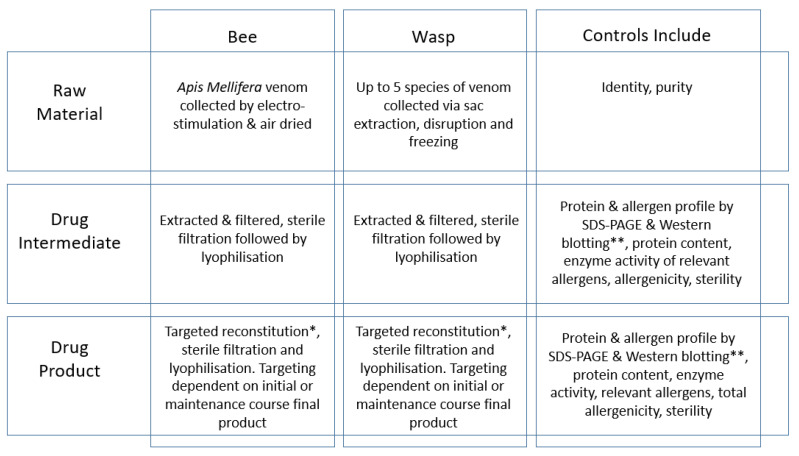
Manufacturing steps for Venomil^®^ with reference to in-process controls. * The target for reconstitution is to fulfil the full drug product specifications (e.g., total allergenicity, enzyme activity and protein content) following the product processing. ** Protein profiling (SDS-PAGE) and allergen profiling (SDS-PAGE and Western blotting) are performed against a controlled, representative in-house reference preparation. Further characterization is performed by proteomics analysis.

Venomil^®^ is filled and lyophilized to provide two different strengths to allow different posologies for the initial course (550 µg) and maintenance course (120 µg). At all stages of the manufacture process, in-process controls are performed to ensure consistent batch-to-batch quality criteria (Figure 1).

## 3. The Molecular Story

Knowledge of Hymenoptera (wasp and honeybee) venoms and their individual allergens is important to understand the mechanisms of venom allergy. Understanding the contributions of certain major or minor allergens and their composition in a venom preparation in relation to the allergic status of the patient is critical to achieving therapeutic success [30,31].

Hymenoptera venoms are complex mixtures of different substances such as amines, peptides and proteins. Most of the venom proteins have enzymatic activity and are responsible for allergic sensitization, allergic symptoms and therapeutic success. The identification of previously unknown allergens in honeybee and wasp venoms using comprehensive proteomic data and genomic information is considered an important element to help augment the effectiveness of immunotherapy [32].

Around 30–40% of patients with insect venom allergy display IgE antibodies that react with venoms from both honeybees and *Vespula* [33]. This may indicate that these patients are indeed sensitized to both venoms or that the double positivity may be the result of cross reactivity based on sequence homology of allergens found in both honeybee and *Vespula* venom. The allergens phospholipase A2 (Api m 1), acid phosphatase (Api m 3), mellitin (Api m 4) and icarapin (Api m 10) are present in honeybee venom but not in *Vespula* venom; and phospholipase A1 (Ves v 1) and antigen 5 (Ves v 5) are only found in *Vespula* but not in honeybee venom [14,15,16,17,18,21,32,34]. These allergens are, therefore, frequently termed marker allergens or species-specific allergens, since specific IgE (sIgE) detection against these allergens allows for better discrimination between honeybee and wasp venom sensitization. Both honeybee and wasp venoms contain hyaluronidase (Api m 2 and Ves v 2), dipeptidyl peptidase IV (DPP IV), (Api m 5 and Ves v 3) and vitellogenins (Api m 12 and Ves v6) [35,36] that show high degrees of sequence identity and similarity and are frequently termed homologous or cross-reactive allergens. sIgE detection to these allergens does not allow for safe discrimination between honeybee venom and *Vespula* venom sensitization [30]. See Table 2 for descriptions of the characteristics of major allergens found in honeybee and wasp venom.

Phospholipases are hydrolases that catalyze the cleavage of fatty acids from phospholipids in cell membranes, and therefore, these enzymes play important roles in bee and wasp venoms’ toxic mechanisms. There are four major classes of phospholipases, A, B, C and D, which catalyze different reactions. For example, phospholipase A1 can catalyze cleavage at the sn1 position and A2 at the sn2 position [31]. The most abundant phospholipase in honeybee venom is phospholipase A2 (Api m 1). The rate of sensitization to phospholipase A2 in different studies of allergic patients ranges from 57 to 97% [9]. Hyaluronidase enzymes are able to degrade hyaluronic acid (hyaluronan), the most abundant glycosaminoglycan in vertebrates, promoting the spread of venom in the body. There are allergens in Hymenoptera venoms that are part of this enzyme class, such as the honeybee Api m 2, *Vespula* spp. Ves v 2 and *P. dominula* Pol d 2 [9]. In the acid phosphatase group, the only allergen is Api m 3 in honeybee venom, but this enzyme can be found in other Hymenoptera species (bumblebee). Acid phosphatases cleave phosphoryl groups from their substrates. The rate of sensitization to Api m 3 ranged from 28 to 63% in different studies of honeybee venom-allergic patients [31]. Dipeptidyl peptidases IV (DPP IV) are enzymes that can cleave N-terminal dipeptides from polypeptides, activating or inactivating substrates. This classification includes the allergens Api m 5, Ves v 3 and Pol d 3 for honeybee, yellow jacket and European paper wasps, respectively [31]. Icaparin (Api m 10) is a major allergen in honeybee venom but with low abundance, and is an unstable protein with unspecified function. In total, 35–73% of honeybee venom-allergic patients showed relevant sensitization to this low abundant allergen [14]. Antigen 5 proteins are part of the CAP (cysteine-rich secretory proteins, antigen 5 and pathogenesis-related 1 proteins) superfamily. These proteins are important major allergens for the majority of Vespoidea species, and sensitization to Ves v 5 is present in 82–98% of wasp venom-allergic patients [9,31].

### Api m 10 Stability in Venomil^®^ and Its Potential Role for Therapeutic Success

**Figure 2:**
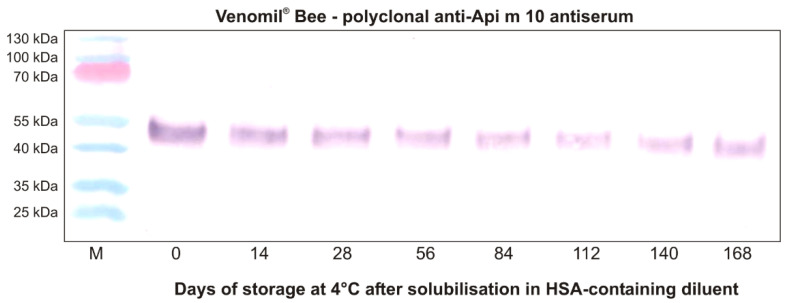
Stability of Api m 10 in Venomil^®^ Bee during long-term storage in solution at 4 °C. Venomil^®^ Bee was solubilized in the product-specific HSA-containing diluent and stored at 4 °C. Aliquots were taken at the indicated time points and stored at −20 °C until final analysis. The samples were analyzed for their Api m 10 content using a rabbit polyclonal Api m 10-specific antiserum, as described previously [19]. HSA, human serum albumin; M, molecular weight marker (Figure and supplementary file provided by Blank S. & Grosch J).

Previous analyses have demonstrated that Api m 10 is an unstable molecule that shows a tendency to degrade in solution [19,36]. However, the use of a diluent containing human serum albumin (HSA) and phenol, used for reconstitution of Venomil^®^, showed a stabilizing effect [19]. Nevertheless, these observations have raised the question of whether the unstable nature of Api m 10 might affect the content of the intact allergen during long-term storage after reconstituting the freeze-dried venom with the diluent. A very recent analysis has addressed Api m 10 stability in Venomil^®^ Bee after solubilization and storage for several months (Figure 2 and Supplement Method S1). First of all, this analysis confirmed the presence of Api m10 in Venomil^®^ Bee in easily detectable amounts, as shown previously [19]. Furthermore, the analysis demonstrated for the first time the presence of intact Api m 10 in Venomil^®^ Bee, reconstituted with the product-specific HSA-containing diluent and stored at 4 °C, through the entire observation period of approximately 6 months. Nevertheless, the content of intact Api m 10 decreased slightly over time. This observation may support the recommendation to use the 120 µg Venomil^®^ vials for maintenance injections, particularly for patients with relevant Api m 10 sensitization.

Allergens found in low abundance in HBV (Api m 3, Api m 5 and Api m 10) have been demonstrated to play an important role as sensitizing allergens, and must be classified as major allergens, as more than 50% of honeybee venom-allergic patients display IgE-reactivity to them [16]. A 2014 study of sensitization profiles of HBV allergic patients found that IgE to Api m 3 and/or Api m 10 was detected in up to 68% of patients [39]. Despite the importance of these low abundant allergens, therapeutic venom preparations may have underrepresentative amounts of Api m 3, Api m 5 and Api m 10 [12]. This allergen underrepresentation was further confirmed by testing allergen sIgG4 responses to different honeybee venom allergens (Api m 1, 2, 3, 4 and 10) in sensitized patients. No or low IgG4 induction was observed in response to Api m 3 and Api m 10 [39]. In contrast, a study from 2016 included a retrospective analysis of sensitization profiles in honeybee venom-allergic patients and their treatment outcomes. In this study, a semi-quantitative analysis was performed to determine the Api m 10 content in therapeutic HBV preparations. Using immunoblotting, it was shown that all HBV preparations contained underrepresentative amounts of of Api m 10 [13]. The levels of specific IgE to Api m 10 were significantly increased in the non-responders (60% of the sIgE to whole HBV directed to Api m 10), suggesting that patients in whom more than 50% of the sIgE to whole HBV was directed against Api m 10 may have higher risk of failure of HBV immunotherapy [13]. However, the study only included a limited number of patients’ sera, warranting further investigations. Importantly, a 2017 study performed by Blank et al. utilizing a novel polyclonal anti-Api m 10 antiserum demonstrated the presence of Api m 10 in several but not all commercial formulations [19]. The Paul Ehrlich Institute (PEI) performed a qualitative identification of Api m 10 in HBV therapeutic products using high definition mass spectrometry (HDMS). The results were presented at the congress of the European Academy of Allergy and Clinical Immunology (EAACI) 2018, demonstrating the detection of Api m 10 in all 19 venom immunotherapy formulations [40,41]. However, it is important to highlight the difference between HDMS and antibody-based Western blot analysis: the latter is reliant on intact, not degraded allergen, whereas HDMS merely detects peptide fragments [14]. The choice of method in this particular instance is highly relevant, given the stability profile of Api m 10, for example.

Meanwhile, a RT-PCR study revealed that there are at least nine to eleven additional Api m 10 transcript isoforms expressed in venom glands of honeybees. This suggests that HBV allergic patients might display different IgE reactivity to different Api m 10 variants [42]. However, one study analyzed the IgE reactivity to different isoforms in honeybee venom-allergic patients sera and reported that most of the isoforms did not display IgE reactivity. Only those with similarities to variants 1 and 2 displayed the highest reactivity (i.e., isoforms 3 and 4) [14,42]. Interestingly, variant 1 and 2 only differed in a stretch of 5 amino acids, resulting from alternative splicing [14]. Recently, a 2020 study identified a major IgE epitope of HBV allergen Api m 10 that was found to be recognized by all sera from HBV-allergic patients sensitized to Api m 10 [43].

In a study of component-resolved diagnosis of venom allergy, IgE reactivity to Api m 3, Api m 10 or both was detected in 68% of patients, and Api m 10 IgE represented the only HBV allergen-specific IgE detected in 5% of patients [16]. Different sensitization studies have found a wide range of Api m 10 IgE reactivity levels, from 35% to 75% [12,13,14,15,16,17,18,31]. It should be noted that the hypothetical or actual links between treatment failure and commercial venom therapy formulations in which Api m 10 is underrepresented are limited to small groups of subjects or patient sub-groups. In any case, it is certainly not wrong to postulate that the sensitizing allergen should be represented within the therapeutic extract. The composition of Venomil^®^ mirrors the natural source venom and includes all major allergens for wasps and bees—including Api m 3, Api m 5 and Api m10 with batch-to-batch consistency [19].

## 4. Clinical Experience with Venomil^®^

Venomil^®^ has been in active clinical use since the early 1980s. In those four decades, a number of studies on its use in patients were published. Table 3 provides a summary of clinical experience with Venomil^®^ and products with shared manufacturing profiles, i.e., Hollister–Stier-produced Albay^®^ [44,45]. Additionally, details of the pivotal clinical trial for market authorization in the US, performed by Bayer/Hollister–Stier [46] in 1978–1981, are included. Thirteen different studies with a total of 1723 patients were identified.

### 4.1. Safety

Nine studies included detailed safety data. To infer safety data from the studies, the number and grades of recorded adverse reactions to Venomil^®^ were recorded in relation to the number of patients treated. This allowed us to give estimated rates of adverse reactions by grade of severity.

All publications noted good tolerability of the Venomil^®^ treatment. The studies demonstrated high rates of local adverse drug reactions (ADRs) (in 100% of patients where specified), and moderate rates of systemic ADRs (between 1.8% [47] and 30% [46]), which are generally in line with data published on the expected rates of systemic adverse reactions in VIT [48].

It is of note that all currently recommended treatment and up-dosing regimens are covered by this safety data (i.e., 2-day ultra-rush [46]; 3-day ultra-rush [49]; 5-day rush [49]; 16-week conventional [50]). It appears there are no obvious advantages or disadvantages in tolerability for any of the specific regimens. In addition, several studies examined experimental posologies and treatment protocols, which were also well tolerated.

### 4.2. Efficacy

Seven studies included detailed efficacy data. A variety of endpoints, including sting challenge, field sting reports, biomarkers and quality of life surveys were used in the studies. In order to infer efficacy rates, we only used data from sting challenges or field sting reports. For comparability, we grouped results into patients with “complete protection” (i.e., no systemic reaction to the sting event) and “partial protection” (i.e., systemic reaction to a sting event of lower grade than before VIT) where possible. “Protection” was used in studies that did not differentiate between no reaction and lower reactions than before VIT. It must be noted that the diverse design of the studies included introduced possible selection bias and reporting bias.

Overall, patients treated with Venomil^®^ showed good protection from systemic reactions to sting events. Partial protection was achieved in between 87.7% [47] and 100% [44,51,52,53,54] of patients. Complete protection was achieved in between 73.7% [51] and 88.7% [46] of patients.

Interestingly, efficacy rates do not appear to differ significantly between honeybee and wasp venom-allergic patients. Complete protection to sting events was achieved in between 71.4% [55] and 75.0% [51] of honeybee patients, and in between 73.3% [51] and 100% [54] of wasp venom-allergic patients. Partial protection was achieved in between 85.7% [47] and 100% [44,50,51,52,53,55] of honeybee venom-allergic patients, and in between 88.2% [47] and 100% [44,51,52,54] of wasp venom-allergic patients. This observation is discordant with a general assumption of reduced efficacy in honeybee VIT [56,57]. While it is not possible to verify this without higher quality, randomized head-to-head trials, this may be a result of the more complete allergen content in non-purified extracts [19].

The clinical use of Venomil^®^ has been established over a close to 40 year period. While double blind placebo-controlled trials are very difficult to implement for this indication [58] and are currently not available, the available data at the point of market authorization together with clinical experience show favorable safety and tolerability profiles, and indications of a good efficacy profile of the treatment.

## 5. The Choice of Depot for Long Term VIT: Considerations

The concept of depot-adjuvanted AIT was originally designed to improve tolerability and the overall safety profile of the therapeutic application of highly allergenic extracts. It was also demonstrated in the 1970s that applying AIT in this way led to the induction of an allergen-specific IgG response [61]. However, while alum offers a depot function and is considered a potent adjuvant, its immunological profile is better understood today as having an overall Th2 bias, which is in discord with the goal of AIT. Where VIT is concerned, there are indeed a number of studies comparing an aqueous extract with alum depot formulations provided by one manufacturer. Furthermore, one other formulation contains a non-inflammatory polysaccharide adjuvant (immunomodulator) [61,62].

Aluminum salts are used in the majority of s.c. depot AIT formulations [63]. Injections in AIT and VIT are administered via the s.c. route, unlike general vaccination where the i.m. route is preferred. Data on the persistence of aluminum depots at s.c. injection sites are extremely sparse. One study extrapolated from rat experiments to man, suggesting that “*aluminum-containing adjuvant would be retained at the s.c. dose site for up to 37 years*” [64]. The potential of aluminum to accumulate and its safety implications are current topics of discourse in AIT [65,66,67], and it is of long-standing concern that regulators only defined a threshold for administration of aluminum per single injection but have thus far neglected cumulative dosing regimens [65].

The Paul Ehrlich Institute (PEI) acknowledged important gaps in scientific information, and initiated a research project related to the “toxicokinetic modelling of aluminum exposure from adjuvants in medicinal products” [67]. A number of publications resulted from this initiative; however, the project has not yet been finalized or published to our knowledge. In their recent publication, the regulators extrapolated from rat experiments to a 3-year perennial s.c. AIT posology involving 36 maintenance doses, each containing up to 1250 μg aluminum, resulting in a cumulative bone aluminum increase of 1–2 μg/g wet weight, which is considered “*substantial but without clinical relevance*” for adults [67].

While regulators continue to assess potential aluminum accumulation and toxicity by AIT or VIT, other stakeholders, such as the European Academy of Allergy and Clinical Immunology (EAACI), have published their opinions following the precautionary principle: “*Although the European Medicines Agency (EMA) had no safety concerns regarding aluminum toxicity from their pharmacovigilance review of aluminum hydroxide in standard AIT, high dose VIT and lifelong therapy has not been specifically evaluated. As a precaution, where life-long therapy is planned it can be undertaken with aqueous preparations. If a 200 μg dose is required for maintenance, half can be given as an aqueous preparation*” [26].

After considering the above, the question might arise of why a depot formulation in VIT remains desirable after all, given the fact that efficacies of aqueous extracts and depot formulations are considered similar [26]. There is more than one way to approach this question.

The most prominent reason circulated within the community might be a postulated safety benefit of depot formulations compared to aqueous extracts. This dogma has spread among allergists for decades, but evidence is surprisingly sparse. There are indeed a number of studies comparing an aqueous extract with a depot formulation provided by one manufacturer. Although there are no significant differences in systemic reactions [62], there appears to be a beneficial safety profile related to local adverse events. Comparative studies have been flawed by comparing not only aqueous extracts with depot formulations but different posologies in parallel, such as comparing conventional gradual up dosing over numerous weeks with ultra-rush protocols [68]. The scientific value of such exercises remains questionable.

If safety is not a sound justification for depot formulations, might it be convenience? The impact on quality of life of repeated injections over 5 years (if not life-long) is obvious. The recommended maintenance intervals of AIT are monthly [69]. However, it might be more convenient to prolong those intervals to 6 weeks. This would be within what is recommended by the European guideline for VIT (at least from year two onwards) [26]. However, it needs to be emphasized that there are aqueous venom extracts allowing 6-week maintenance intervals from year two onwards, according to their summaries of product information (SmPC). Thus, 6-week intervals are not a “unique selling point” of depot formulations. Following this thought, a logical question is whether intervals can be prolonged even further. The US Practice Parameters suggest that the maintenance dose can be given at intervals of 4 weeks for the first 12–18 months, then 6 weeks for a year, then 8 weeks for a year and then 12 weeks thereafter, as indicated. In addition, there are publications describing safe and effective use of aqueous VIT extracts over 12-week intervals [55]. Extended long intervals (despite appearing convenient) interfere with the dogma of cumulative dosage driving efficacy of VIT. This is why European guidelines do not recommend longer intervals [26].

Since the benefits and safety of a depot adjuvant are not obvious and clear-cut, it is difficult to conclude a science-based justification. A notable feature, in case a decision is made to select a depot formulation, is that guidance of the respective SmPC needs to be strictly followed.

Perhaps the desire for a depot VIT formulation is more an expression of the current marketing reality. However, taken the above safety considerations around aluminum body burden and VIT into account, most likely the true unmet need would be a depot VIT formulation using an effective, safe and biodegradable depot formulation. The crystalline form of the physiological non-essential amino acid L-tyrosine, MicroCrystalline Tyrosine = MCT^®^, is a Th1-polarizing depot adjuvant that has been in use for many years—more than 9 million injections have been administered, including some to vulnerable populations [70]. The mode-of-action of MCT^®^ was recently described in a state-of-the-art head-to-head adjuvant study [71]. Physico-chemical properties and depot functions are well-documented [72,73]. A recent position paper, authored by an independent taskforce of EAACI members but also of a representative of the PEI stated, “*Since its introduction into AIT in 1970, there are no specific safety concerns known for MCT. It can be anticipated that this fully biodegradable adjuvant will also in future studies not reveal side effects*” [63]. Thus, there is a well-established alternative depot adjuvant in AIT with a long-term superior safety profile in humans making MCT^®^ “*a better adjuvant compared to alum*” [74]. Allergy Therapeutics plc as the patent holder of MCT^®^ and manufacturer of VIT products is currently exploring ways to develop MCT^®^-adjuvanted depot VIT formulations.

## 6. Conclusions and Summary

A wealth of quality and clinical data using venom products derived from shared material sources have been consolidated here. We demonstrated well-established and favorable safety and efficacy profiles from overall treatment outcomes that are considered similar between wasp and bee VIT. Since the composition of Venomil^®^ mirrors that of the natural venom, it includes the allergens for wasps and bees, i.e., Ves v 1, Ves v 2 and Ves v 5 or Api m 1, Api m 2, Api m 3, Api m 5 and Api m 10, with batch-to-batch consistency. This further validates the conclusions about treatment efficacy between broadly comparable products.

Quality differences between products and their impacts on treatment outcomes have been current topics of discussion and further research [75,76]. The lessons here are relevant for other treatment indications where allergens of growing importance exist and have been demonstrated experimentally to be more prone to modifications in downstream manufacture processing steps or changes in natural abundance related to shifts in global or environmental conditions. This highlights the need to continue to advance the molecular basis of understanding their roles with respect to treatment outcomes.

The success rates of VIT are substantially higher compared to AIT, but the standard of care requires longer treatment courses in often-high dose settings. Furthermore, there is a considerably large group of insect venom allergy patients who require life-long VIT. As such, depot adjuvants such as alum, which has the propensity to accumulate, should be reconsidered in a risk–benefit context, where better clarity of added benefit can be explored further. Biodegradable adjuvant platforms, designed to support the immunological effect of the treatment (i.e., Th1-specific), provide a rational option to augment VIT.

## Figures and Tables

**Table 1:** Current market authorization for venom products.

Company	Venoms Covered under Marketing Authorization (MA)	Country in Which Market Authorization Is Held
Bayer	All Hymenoptera venom products	US
Stallergenes	Honeybee and Yellow jacket	France
Allergy Therapeutics *	All Hymenoptera venom products	Germany

* Non-registered formulations of the product known by Allergy Therapeutics as Venomil^®^ such as Albay™ Hymenoptera venom products are commercially available in UK, Spain, Italy, Greece, Estonia, Lithuania, Albania, Latvia and Switzerland. MA, market authorization.

**Table 2:** Characteristics of the major allergens.

Biochemical Name	Venom	Allergen Nomenclature	MW	Potential N-Glycosylation	Allergenicity
**Phospholipases**					
A2	Honeybee	Api m 1	16 kDa	1	An in-vitro study using flow-cytometry analysis, showed an increase of basophil CD203c in response to Api m 1 in 9 of 13 patients with bee allergy. Specific IgE levels to Api m 1 increased in 4 patients allergic to bee venom (quantified by fluorescence immunoassay (UniCAP^®^); 5 patients tested) [37]. The sensitization rate to Api m 1 is reported to range from 57–97% in honeybee venom-allergic patients in different cohorts [31]. PDB structure: 1POC Uniprot: P00630
A1B	Wasp	Ves v 1	34 kDa	0	In the same in-vitro study as above, 17 patients with allergy of wasp venom presented upregulation of CD203c basophil response when stimulated with purified Ves v 1 [37]. IgE sensitization to Ves v 1 varies from 39–66% as per different populations of wasp allergic patients and is reported to increase in wasp/honeybee double-sensitized patients [31]. UniProt: P49369
**Hyaluronidases**					
	Honeybee	Api m 2	44 kDa	3	An in-vitro study demonstrated that all 13 patients with bee allergy responded to purified Api m 2 by increasing levels of basophil activation marker CD203c. Specific IgE levels were increased in 5 patients that reacted with Api m 2 using RAST in 5 patients allergic to bee venom (39 patients tested) [37]. Sensitization rates range from 28–60% in different study populations (28–55 in honeybee venom-allergic patients and 45–60% in honeybee/wasp venom double-sensitized patients) [31]. PDB structure: 1FCQ UniProt: Q08169
	Wasp	Ves v 2	38 kDa	4	Of 35 patients allergic to wasp venom, 26 patients presented upregulation of CD203c basophil response when stimulated with purified Ves v 2 [37]. Around 10–15% of patients with wasp allergy are estimated to have IgE antibodies against Ves v 2 and peptide-specific cross-reactivity with Api m 2 [31]. PDB structure: 2ATM Uniprot: P49370 & Q5D7H4
Acid phosphatases	Honeybee	Api m 3	43–49 kDa	2	Recombinant allergen Api m 3 showed immunoreactivity to specific IgE antibodies in pooled serum by Western blot and 37% in individual sera by ELISA in honeybee venom-sensitized patients [38]. The sensitization rate is reported to range from 28–63% in different studies of honeybee venom-allergic patients [31]. Uniprot: Q4TUB9
Dipeptidyl peptidase IV	Honyebee	Api m 5	100 kDa	6	IgE reactivity to Api m 5 was detected in 58.3% of 144 honeybee venom allergy patients [16]. The sensitization rate is reported to range from 16–70% in different studies of honeybee venom-allergic patients [31]. Uniprot: B2D0J4
Icarapin variant 2	Honeybee	Api m 10	50–55 kDa	2	IgE reactivity presented in 50% of 84 honeybee venom-sensitized patients [12]. The sensitization rate varies from 35–75% in honeybee venom-allergic patients [31]. Uniprot: Q1HHN7
**Antigen 5**	Wasp	Ves v 5	23 kDa	0	Of 26 patients allergic to wasp venom and 8 patients allergic to bee and wasp venom (24 tested patients), 27 showed upregulation of CD203c expression in basophils in response to Ves v 5 [37]. The sensitization rate of Ves v 5 is reported to range from 82–98% in wasp venom-allergic patients [31]. PDB structure: 1QNX UniProt: Q05110
**PDB**: Protein Data Bank; **UniProt**: Universal Protein Resource (comprehensive database of protein sequence).					

**Table 3:** Clinical data.

Publication	Species	Class	Patients	Implied ADR Rates	Implied Efficacy Rate	Notes	Reference
**Döring et al., 1994**	Honeybee + Wasp	Retrospective NIS	612	100% local ADRs 3.9% ADRs grade I+ 0.9% ADRs grade II+	99.3% protection (100% HB, 99.0% Wsp)	Analysis of 14 years of patient data from private practice	[50]
**Baenkler et al., 2005**	Honeybee + Wasp	Non-controlled IIT	176	N/A local ADRs 14.2% systemic ADRs 1.7% ADRs grade II+	89.3% complete protection (71.4% HB, 88.7% Wsp, 100% HB + Wsp) 98.7% partial protection (100% HB, 98.1% Wsp, 100% HB + Wsp)	Venomil and Reless; Off-label continuation course using 6-month-intervals after month 9.	[55]
**Jung et al., 2002**	Honeybee + Wasp	NIS	50	N/A local ADRs 12.0% systemic ADRs 0% ADRs grade II+	73.7% complete protection (75.0% HB, 73.3% Wsp) 100% partial protection	Evaluation of biomarkers (sIgE, skin tests) for treatment control	[51]
**Lee et al., 2005**	Wasp	NIS	50	100% local ADRs 10% systemic ADRs 2% ADRs grade II+	N/A	Tolerability study of rush- and ultra-rush posologies	[49]
**Lohse et al., 2005**	Honeybee + Wasp	Non-controlled IIT	36	N/A	100% protection	Evaluation of a “super rush dose regimen” as treatment control and possible booster after 3–5 years of VIT	[52]
**Münstedt et al., 2010**	Honeybee	Retrospective NIS	43	N/A	97.7% complete protection 100% partial protection	Survey and clinical follow-up of VIT-treated beekeepers; 80% Venomil patients, 20% Reless; high chance of selection bias	[53]
**Lee et al., 2008**	Wasp	Non-controlled IIT	11	N/A	100% protection	Basophil activation test as treatment control; sting challenge after 1 year	[54]
**Roesch et al., 2008**	Honeybee + Wasp	Retrospective NIS	137	N/A	80% complete protection 87.7+% partial protection	Survey of VIT-treated patients	[59]
**Stoevesandt et al., 2019**	Honeybee + Wasp	Retrospective NIS	44	N/A local ADRs 6.8% systemic ADRs	N/A	Tolerability of Venomil vs. ALK lyophilisiert/depot SQ	[60]
**Becker et al., 2020**	Honeybee + Wasp	Retrospective NIS	114	N/A local ADRs 1.8% systemic ADRs	87% partial protection (85.7% HB, 88.2% Wsp)	Analysis of 16 years of patient data from one university pediatrics department	[47]
**Bayer Inc. 1982**	Honeybee + Wasp	Prospective Open-Label Clinical Trial	114	N/A local ADRs 30% systemic ADRs	88.7% complete protection 98.3% partial protection	Licensing study for Safety and Efficacy in the US; sting challenge after reaching maintenance dose	[46]
**Nataf et al., 1984**	Honeybee + Wasp	Prospective Open-Label Clinical Trial	52	N/A local ADRs N/A systemic ADRs 1.9% ADRs grade II+	100% protection	Evaluation of four-day rush initiation treatment	[44]
**Birnbaum et al., 1993**	Honeybee + Wasp	Prospective Open-Label Clinical Trial	284	100% local ADRs 12.0% systemic ADRs	N/A	Evaluation of three different rapid initiation treatments	[45]

NIS—non-interventional study; IIT—investigator initiated trial.

## Data Availability

Non applicable.

## Supplementary Materials

The following are available online at https://www.mdpi.com/article/10.3390/toxins13090616/s1, Method S1. Analysis of Api m10 content in Venomil^®^ Bee during long-term storage.
