# Supplementary material for: Venom Immunotherapy: From Proteins to Product to Patient Protection
Source: Toxins (Basel). 2021 Sep 1;13(9):616. doi: 10.3390/toxins13090616 (PMC8470233; doi:10.3390/toxins13090616)
Supplement: Supplementary file 1 [file toxins-13-00616-s001.zip › toxins-1283470-supplementary.pdf]

Supplementary Materials: Venom Immunotherapy: From Proteins to Product to Patient Protection

Martin Feindor, Matthew D. Heath, Simon J. Hewings, Thalia L. Carreno Velazquez, Simon Blank, Johannes Grosch, Thilo Jakob, Peter Schmid-Grendelmeier, Ludger Klimek, David B.K. Golden, Murray A. Skinner and Matthias F. Kramer

Method S1. Analysis of Api m10 content in Venomil® Bee during long-term storage

Freeze-dried Venomil® Bee (Allergy Therapeutics, Worthing, UK; Lot A90526 LR) was dissolved in the product-specific HSA-containing diluent (Allergy Therapeutics, Lot A90487 LV) to a final concentrations of 650 µg/mL. This higher concentration compared to that used for VIT injections in the clinic was chosen due to the detection limit of the Western Blot. However, this higher concentration should have only minor effects on allergen degradation due to the high amounts of stabilizing HSA in the solution. Reconstituted Venomil® Bee was stored at 4°C in the original glass vial. Samples of 25 µL were drawn out of the stock on day 0, 14, 28, 56, 84, 112, 140 and 168. Samples were immediately mixed with 5 µl of reducing loading buffer and incubated for 10 minutes and 90°C before frozen away and stored at -20°C.

28 µl of thawed samples, which translates to approximately 15 µg HBV, were loaded onto freshly casted Tris-Tricine gels and subsequently blotted onto nitrocellulose membranes (Thermo Fisher Scientific, Schwerte, Germany). Membranes were blocked for 1 h with 40 mg/mL non-fat dry milk powder (AppliChem, Darmstadt, Germany) in PBS (Thermo Fisher Scientific) at RT, before incubation over night at 4°C with polyclonal Api m 10-specific rabbit antiserum (1:1000 in 20 mg/mL nonfat dry milk powder in PBS) (Davids Biotechnology, Regensburg, Germany), generated by immunisation of rabbits with recombinant Api m 10 as described previously [20]. After washing with PBS for 3 times, Api m 10-specific antibodies were detected for 1 h at RT with polyclonal goat anti-rabbit IgG antibody conjugated to alkaline phosphatase (1:5000 in 20 mg/mL nonfat dry milk powder in PBS) (Sigma-Aldrich, Taufkirchen, Germany). Membranes were washed 3 times with PBS, before adding nitrotetrazolium blue chloride / 5-bromo-4-chloro-3-indoyl phosphate (AppliChem) as substrates for visualization.
